# A systematic review of hepatic tuberculosis with considerations in human immunodeficiency virus co-infection

**DOI:** 10.1186/s12879-015-0944-6

**Published:** 2015-05-06

**Authors:** Andrew J Hickey, Lilishia Gounder, Mahomed-Yunus S Moosa, Paul K Drain

**Affiliations:** University of Maryland School of Medicine, 655 W. Baltimore Street, Baltimore, MD 21201 USA; Department of Infectious Diseases, Nelson Mandela School of Medicine, University of KwaZulu-Natal, Durban, South Africa; Department of Virology, National Health Laboratory Service, Inkosi Albert Luthuli Central Hospital, Durban, South Africa; Division of Infectious Diseases, Massachusetts General Hospital, Harvard Medical School, Boston, USA; Medical Practice Evaluation Center, Department of Medicine, Massachusetts General Hospital, Harvard Medical School, Boston, USA

**Keywords:** Tuberculosis, Liver, Extrapulmonary tuberculosis, HIV/AIDS

## Abstract

**Background:**

*Mycobacterium tuberculosis* (TB) infection of the liver, known as hepatic TB, is an extrapulmonary manifestation of TB. Hepatic TB has become more prevalent, likely as a result of the human immunodeficiency virus/acquired immunodeficiency syndrome (HIV/AIDS) epidemic. We sought to review case series to characterize the epidemiology, pathophysiology, clinical features, diagnosis, and treatment of hepatic TB and to comment on the impact of HIV co-infection on these characteristics.

**Methods:**

We conducted a systematic literature search in PubMed and ScienceDirect for articles pertaining to hepatic TB with human subjects from 1960 to July 2013.

**Results:**

We obtained data on 618 hepatic TB patients from 14 case series. The most common reported signs and symptoms were hepatomegaly (median: 80%, range: 10-100%), fever (median: 67%, range: 30–100), respiratory symptoms (median: 66%, range: 32-78%), abdominal pain (median: 59.5%, range: 40-83%), and weight loss (median: 57.5%, range: 20-100%). Common laboratory abnormalities were elevated alkaline phosphatase and gamma-glutamyl transferase. Ultrasound and computerized tomography (CT) were sensitive but non-specific. On liver biopsy, smear microscopy for acid-fast bacilli had a median sensitivity of 25% (range: 0-59%), histology of caseating granulomas had a median sensitivity of 68% (range: 14-100%), and polymerase chain reaction for TB had a median sensitivity of 86% (range: 30-100%). Standard anti-tuberculous chemotherapy for 6 to 12 months achieved positive outcomes for nearly all patients with drug-susceptible TB.

**Conclusions:**

Clinicians in TB-endemic regions should maintain a high index of suspicion for hepatic TB in patients presenting with hepatomegaly, fever, respiratory symptoms, and elevated liver enzymes. The most sensitive imaging modality is a CT scan, while the most specific diagnostic modality is a liver biopsy with nucleic acid testing of liver tissue samples. Upon diagnosis, 4-drug anti-TB therapy should promptly be initiated. HIV co-infected patients may have more complex cases and should be closely monitored for complications.

## Background

*Mycobacterium tuberculosis* (TB) usually infects the lungs, called pulmonary TB, but can infect almost any organ in the body, causing an extrapulmonary infection. TB infection of the liver, called hepatic TB, is an extrapulmonary manifestation of an active infection. The first recorded case of hepatic TB was reported in 1858 by Dr. John Syer Bristowe, an English physician [1]. In 1905, more than 20 years after Koch’s discovery of the TB bacillus, Drs. Rolleston and McNee had classified hepatic TB into miliary (disseminated) and local (isolated) forms [2]. Since then, several case reports and few limited case series have described patients with both forms of hepatic TB, but there has been no systematic review of hepatic TB.

The incidence of TB underwent a resurgence in the 1980s, and the World Health Organization (WHO) estimates that 8.7 million people develop active TB disease and 1.4 million die from TB annually [3,4]. While the incidence of active TB likely peaked in 2004, the proportion of extrapulmonary TB cases continues to rise [5,6]. In the United States, the proportion of extrapulmonary TB cases nearly tripled from 7.6% in 1962 to 21% in 2006 [6]. The HIV/AIDS pandemic, coupled with poor health care delivery in many resource-limited countries, has fueled the resurgence of TB [7]. High TB incidence rates occur where HIV is most prevalent, and the immunosuppression caused by HIV leads to a reactivation of latent TB [8]. HIV/AIDS has also contributed to the relative rise in extrapulmonary TB rates [9], as the risk of extrapulmonary TB increases with decreasing CD4 counts [10]. Over 50% of HIV and TB co-infected people present with extrapulmonary involvement, which includes hepatic TB [11].

A clearer understanding of hepatic TB will help clinicians with diagnostic and management decisions in order to improve patient outcomes. There have been no prior systematic reviews of hepatic TB to facilitate this understanding; therefore, the aim of this systematic review is to synthesize the existing data on the epidemiology, pathophysiology, clinical features, diagnosis, and treatment of hepatic TB, and to highlight additional considerations in HIV co-infection.

## Methods

### Literature search and inclusion criteria

**Figure 1:**
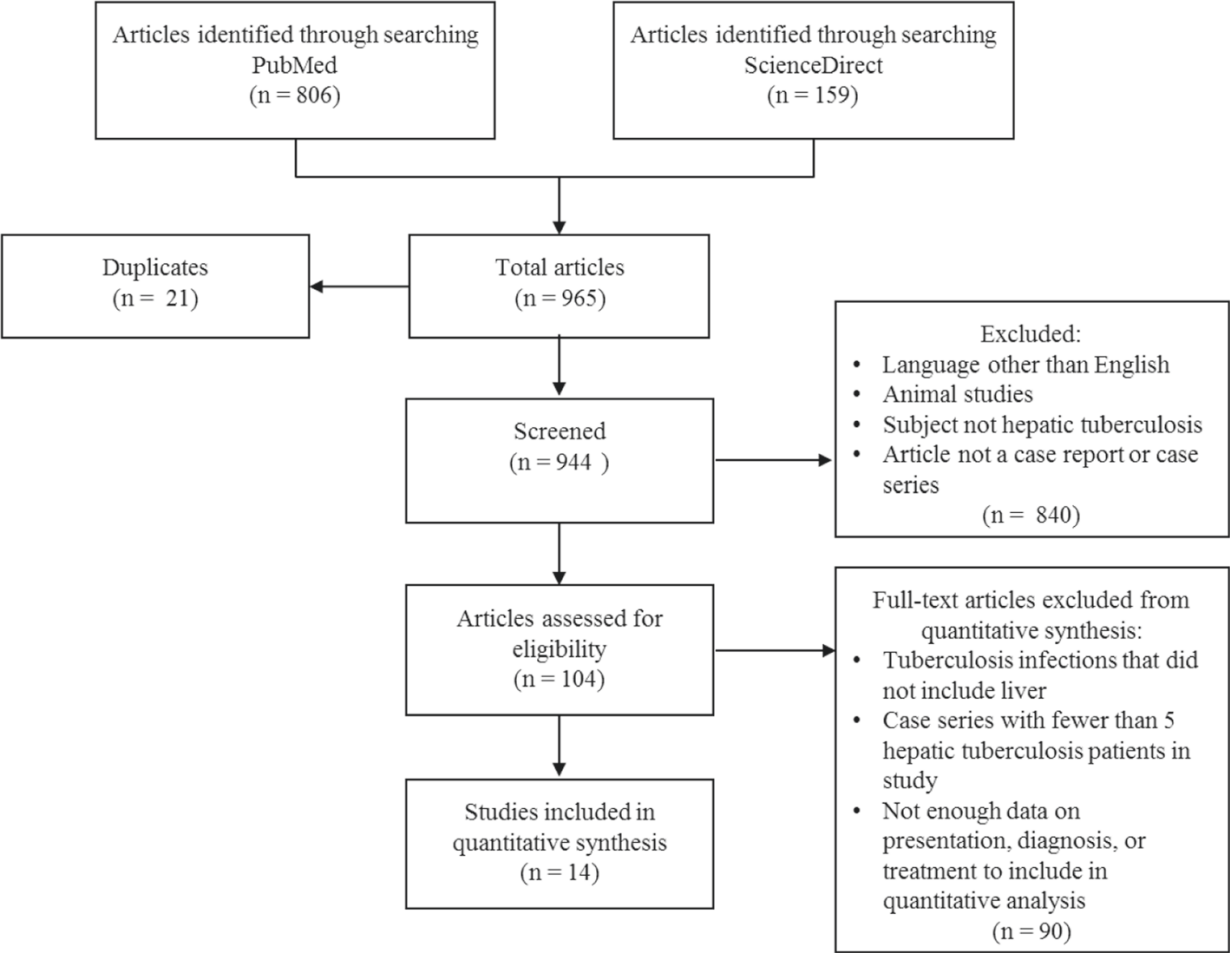
Flow diagram of literature search and study selection.

We conducted a systematic literature search in PubMed and ScienceDirect for articles pertaining to hepatic TB (Figure 1). The primary search term used was “hepatic tuberculosis.” We included all articles published between 1960 and July 2013. There were a total of 965 hits (806 in PubMed, 159 in ScienceDirect). Of these, there were 21 duplicate articles which were removed, leaving 944 to be screened. Of the 944 articles screened, 840 were excluded under the following criteria: published in language other than English, non-human animal study, subject of study was not hepatic TB, and study was not a case report or case series. The remaining 104 articles were assessed for eligibility for quantitative analysis. For the purpose of our study, we included only hepatic TB case series, and we defined a case series as having five or more patients diagnosed with hepatic TB. We also required that the case series have sufficient data to perform quantitative analysis on clinical presentation, diagnosis, and treatment of hepatic TB. Of the 104 articles assessed for eligibility, 14 met our inclusion criteria for quantitative analysis, and the remaining 90 were excluded for the following reasons: patients had tuberculosis infections that did not include the liver, fewer than 5 patients with hepatic TB in study, and insufficient data on presentation, diagnosis, and treatment to perform quantitative analysis. We also reviewed references from the selected manuscripts to obtain additional information relevant to the epidemiology, pathophysiology, clinical features, diagnosis, and treatment of hepatic TB. These references were published any time prior to July 2013.

### Data analysis

From identified manuscripts and case series, we abstracted data on the epidemiology, pathogenesis, clinical features, diagnosis, and treatment. The method of analysis varied by the aspect of hepatic TB being examined and the data available from the manuscripts. The epidemiology of hepatic TB was evaluated by abstracting the year of the study, geographic location, proportion of male patients, and mean age of cases. The incidence and mortality of hepatic TB were approximated using data available from some of the larger case series. Pathological features were determined by calculating the proportion of total hepatic TB cases that were miliary hepatic TB and those that were local hepatic TB. Where available, we also abstracted information on pathogenesis from the introduction and discussion sections, as well as from other manuscripts referenced in the studies. To determine the key clinical features of hepatic TB, we determined the most common signs and symptoms by calculating the proportion of patients with each sign and symptom from each of the case series, and we then presented the median value and range of these percentages. For assessing liver function test abnormalities, the median values from each case series were calculated, and the range of these medians, rounded to the nearest 50 U/L, were reported. The diagnosis of hepatic TB was evaluated by calculating the median proportion and range of positive diagnostic findings on chest radiograph, abdominal ultrasound, abdominal computerized tomography (CT), and liver biopsy. The treatment of hepatic TB cases was not provided in all cases series. When treatment information was provided, the regimen, number of patients receiving that regimen, median treatment duration, and outcomes were assessed and tabulated.

### Ethics

This study was a systematic review of the medical literature and did not involve any human subjects. As such, no approval from an ethics committee was required.

## Results and Discussion

Our search identified 14 manuscripts, describing 618 cases of hepatic TB that occurred before or during the HIV/AIDS epidemic. The global distribution of reported hepatic TB cases was concentrated in Sub-Saharan Africa and Southeast Asia, which is similar to the distribution of pulmonary TB (Table 1) [12]. Approximately 79% of all reported hepatic TB cases were due to systemic dissemination of bacilli, called miliary hepatic TB. However, several cases of local hepatic TB, which are isolated infections of the liver, have been reported in South Africa, the Philippines, and India [11,13-16]. Since hepatic TB is underreported in published literature, any regional clustering of described hepatic TB cases may reflect a reporting bias, and the distribution of hepatic TB likely mirrors the global epidemiology of active pulmonary TB cases.

**Table 1:** **Reported case series of hepatic tuberculosis**

**Author**	**Year**	**Location**	**Total (N)**	**Male (%)**	**Age (Mean)**	**Miliary (%)**	**Local (%)**	**Comments**
Hersch [32]	1964	South Africa	200	58	33	90	10	Primarily diagnosed at autopsy
Alvarez & Carpio [29]	1983	Philippines	130	66	11-50^#^	-	-	Two groups: w/ and w/o jaundice
Essop et al. [18]	1984	South Africa	96	-	20-70^#^	90	10	+/− prognostic groups
Maharaj et al. [20]	1987	South Africa	41	59	45	-	-	22-month prospective study
Chien et al. [2]	1994	Taiwan	22	50	48	77	23	Comparison of miliary and local forms
Kok et al. [44]	1999	Brunei	5	80	41	0	100	Brief cases and literature review
Huang et al. [49]	2003	Taiwan	5	60	60	0	100	Retrospective PCR analysis
Vilaichone et al. [45]	2004	Thailand	20	85	35	-	-	10 HIV+, 2 SLE, 8 immunocompetent
Yu et al. [60]	2004	China	12	58	38	33	67	CT, MRI, US findings
Desai et al. [14]	2006	India	7	86	2-65^#^	0	100	2-year retrospective; 1 HIV+
Amarapurkar et al. [76]	2008	India	38	71	38	39	61	Study excluded patients with HIV
Tai et al. [19]	2008	Taiwan	10	50	53	60	40	15-year retrospective study
Hwang et al. [50]	2009	South Korea	12	75	48	67	33	3 immunocompromised (HIV-)
Gounder et al. [30]	2012	South Africa	20	30	34	100	0	All HIV+

PCR = Polymerase chain reaction; HIV = Human immunodeficiency virus; SLE = Systemic lupus erythematosus; CT = Computerized tomography; MRI = Magnetic resonance imaging; US = Ultrasound.

^#^Approximate majority age range.

The prevalence of hepatic TB has been estimated in several studies. An early case series published in 1930 estimated TB liver involvement in 50-80% of patients with advanced pulmonary TB [17]. Over a six-year period from 1978–1984, Essop et al. diagnosed hepatic TB by liver biopsy and/or autopsy in 1.2% (96/8,342) of all patients with active TB in a South African hospital, some of whom might have been HIV-positive [18]. Tai et al. found a similar proportion of hepatic TB (0.8%; 10/1,251) among patients with active TB during a 15-year period in Taiwan [19]. At one South African hospital in 1986, nearly 10% of patients (whose HIV status was unknown or not reported) with unexplained hepatomegaly were diagnosed with hepatic TB by liver biopsy [20]. The incidence of hepatic TB remains unknown, likely due to unfamiliarity of the disease, since historically most hepatic TB was diagnosed upon surgery or autopsy [21]. A conservative estimate of the incidence can be made using data from the studies conducted by Essop et al. and Tai et al., who found hepatic TB in approximately 1% of all active TB cases [18,19]. Applying this percentage to the world’s TB incidence, 8.7 million per year [3], suggests there may be approximately 87,000 new cases of hepatic TB each year.

The proportion of extrapulmonary TB, which includes hepatic TB, has increased in the last 30 years [22], due largely to increases in the global prevalence of HIV/AIDS [11,21-26]. In a study of 164 patients with disseminated TB, hepatic TB was observed in 17.4% (4/23) of HIV-infected people and 4.3% (6/141) of HIV-uninfected people [23]. In an autopsy study on 39 patients who died of HIV complications in Johannesburg, South Africa, TB was the cause of death in 69% (27/39) of cases, and among those, 33% (9/27) had TB cultured from their liver specimen [27]. In an autopsy study from Nigeria, histologic evidence of TB was found in 37.3% (25/67) of liver tissue samples from patients who died of HIV/TB co-infection complications [28]. These studies suggest that hepatic TB may be underdiagnosed and commonly associated with mortality in HIV and TB co-infected patients.

Among HIV-uninfected patients, the case fatality rate of hepatic TB was between 12 and 42% [18,29]. However, Essop et al. reported 95% (35/37) of untreated patients died in their case series [18]. Poor prognostic factors included age <20 years, miliary TB, predisposing factors (treatment with steroids, chronic renal failure, diabetes, systemic lupus erythematosus, and significant alcohol intake), coagulopathy, low prothrombin index, and greater extent of caseation on histology [18]. There was no definitive data on the mortality rate for HIV-infected hepatic TB patients. In our recent study of 20 HIV-infected hepatic TB patients in South Africa, the mortality was 40% despite prompt initiation of standard 4-drug anti-tuberculosis therapy [30]. Although this was comparable to the observed mortality among HIV-uninfected adults, the results are limited by a small sample size.

### Dissemination and pathophysiology of hepatic tuberculosis

Tuberculous bacilli can reach the liver via hematogenous dissemination, generally from the lungs, or by local spread from the gastrointestinal tract (Table 2) [2,3,31,32]. Among reported hepatic TB cases, miliary form accounted for 79% of cases, while local hepatic TB accounted for 21% of cases (Table 1). TB infection of the biliary tree, or biliary TB, is another form of TB infection in the liver and is considered rare [2,33].

**Table 2:** **Contrasting miliary and local hepatic TB**

	**Miliary Hepatic TB**	**Local Hepatic TB**
Prevalence	79%	21%
Infection origin	Lungs	Gastrointestinal system
Dissemination	Hepatic artery	Portal vein
Tubercle size	0.6-2.0 mm in diameter	>2.0 mm in diameter
Location of tubercles	Lobules of liver	Near portal triad
Clinical signs/symptoms	Cough, sputum production, hepatomegaly	Weight loss, hepatomegaly, jaundice
Diagnosis	CT scan; liver biopsy is less helpful	Liver biopsy more helpful
CT findings	Multiple, dispersed, low-density micronodules	Large, low-density nodules with calcification and peripheral enhancement
Treatment	4-drug regimen	4-drug regimen; consider drainage of abscess or surgery

TB = Tuberculosis; CT = Computerized tomography.

Hematogenous dissemination, or miliary disease, from a pulmonary focus is the most common etiology of hepatic TB [32]. The source of miliary dissemination may be from another extrapulmonary site, such as an abdominal lymph node, but this is rare. In miliary hepatic TB, bacilli reach the liver via the hepatic artery [2,32,33]. Miliary hepatic TB is characterized by diffuse seeding of the liver with tubercles ranging from 0.6 to 2.0 mm in diameter situated in the lobules of the liver [32].

In local hepatic TB, which has also been called tuberculoma, macronodular hepatic TB, or pseudotumoral hepatic TB, dissemination primarily occurs via the portal vein from a focus in the gastrointestinal tract [32]. Local hepatic TB is typically characterized by tubercles greater than 2 mm in diameter situated near the portal triad region [2,32]. A TB liver abscess commonly arises from local hepatic TB, but may also occur following miliary hepatic TB [32]. Local hepatic TB tends to cause more hepatocellular damage than miliary hepatic TB [2]. In contrast to miliary hepatic TB, those with local hepatic TB do not generally have evidence of active pulmonary disease [2].

The characteristic histological feature of both miliary and local forms of hepatic TB is the granuloma [11,34]. Hepatic granulomas are due to cell-mediated immunological responses to TB antigens and consist of focal aggregates of macrophages, including Kupffer cells that may coalesce to form Langerhans giant cells with surrounding lymphocytes and fibroblasts [34-37]. Granulomas may be necrotizing or non-necrotizing, and caseating (where necrotic tissue appears cheese-like on gross examination) or non-caseating. Hepatic granulomas in response to TB tend to be necrotizing with central caseation [34]. In three large case series, caseating granulomas were found in 51-83% of hepatic TB patients [18,20,29]. Multiple granulomas may coalesce to form a large tuberculoma, and caseation and liquefaction necrosis of a tuberculoma may lead to a tubercular abscess [11].

TB was the most common etiology of hepatic granulomas in TB-endemic countries [36,38,39]. In Iran, India, and Saudi Arabia, TB was the cause of hepatic granulomas among 51% (37/72), 55% (28/51), and 43% (26/61) of cases, respectively [36,38,39]. In Turkey, TB was among the most common causes of hepatic granulomas (15%) [40]. Therefore, in TB-endemic countries, presence of hepatic granulomas was highly suggestive of hepatic TB.^11^ In low-prevalence TB regions and among patients with no risk factors for TB, non-communicable causes, such as primary biliary cirrhosis or sarcoidosis, may be more common etiologies for hepatic granulomas [35,37].

HIV alters the pathophysiology of hepatic TB. Immunocompromised HIV-infected patients not only have increased susceptibility to TB reactivation and dissemination, but their manifestations of extrapulmonary TB tend to be more severe [8,22]. HIV-infected patients with extrapulmonary TB are also more likely to have concomitant pulmonary TB [8]. An increased risk of dissemination and development of abdominal TB among HIV-infected people may explain their greater risk of developing hepatic TB than HIV-uninfected persons [23]. The isolated local form of hepatic TB is more common in immunocompromised patients [41,42]. In addition, granulomas in AIDS patients are typically absent or poorly formed and lack necrosis due to a dysfunctional immune system [43]. Hepatic TB in HIV-infected and -uninfected patients differs both clinically and pathologically.

### Clinical features of hepatic tuberculosis

Clinical features of hepatic TB are nonspecific, which often leads to a diagnostic delay [23]. Analysis of eleven hepatic TB case series revealed the most common presenting signs/symptoms were hepatomegaly (median: 80%, range: 10-100%), fever (median: 67%, range: 30-100%), respiratory symptoms (median: 66%, range: 32-78%), abdominal pain (median: 59.5%, range: 40-83%), and weight loss (median: 57.5%, range: 20-100%) (Table 3). Other signs included splenomegaly (median: 30%, range: 0-40%), ascites (median: 23%, range: 5-25%), and jaundice (median: 20%, range: 0-60%). Local hepatic TB and miliary TB may differ in presentation. Local hepatic TB may present primarily as diffuse abdominal pain, while patients with miliary hepatic TB may present with acute respiratory symptoms such as a cough, with or without sputum production [2,19,44,45]. Jaundice is also more common in cases of local hepatic TB and biliary TB [32]. Presenting signs and symptoms are similar in HIV-infected and HIV-uninfected patients [11,46-48]. However, as before, HIV-infected patients are more likely to have a concomitant pulmonary TB infection [8].

**Table 3:** **Presenting signs and symptoms of hepatic TB patients**

**Lead author**	**N**	**Frequency of presenting sign/symptom (%)**							
**(Year)**		**Hepatomegaly**	**Fever**	**Respiratory SX**	**ABD pain**	**LOW**	**Splenomegaly**	**Ascites**	**Jaundice**
Hersch (1964) [32]	143	74	97	66	55	83	35	ND	22
Alvarez (1983) [29]	130	96	65	ND	45	55	25	ND	35
Essop (1984) [18]	96	80	70	74	66	ND	40	25	11
Maharaj (1987) [20]	41	95	63	78	46	61	32	24	15
Chien (1994) [2]	22	50	64	32	59	32	18	23	18
Kok (1999) [44]	5	100	ND	ND	80	ND	0	ND	60
Vilaichone (2004) [45]	20	80	100	ND	60	60	40	ND	20
Yu (2004) [60]	12	33	67	ND	83	42	ND	ND	17
Desai (2006) [14]	7	100	100	ND	71	100	ND	ND	29
Tai (2008) [19]	10	10	30	ND	40	20	10	10	0
Gounder (2012) [30]	20	85	ND	40	ND	ND	30	5	35
Median (Range)		80 (10–100)	67 (30–100)	66 (32–78)	59.5(40–83)	57.5 (20–100)	30 (0–40)	23 (5–25)	20 (0–60)

TB = Tuberculosis; SX = Symptoms; LOW = Loss of weight; ABD = Abdominal; ND = Not documented.

The most common abnormalities associated with hepatic TB included elevated alkaline phosphatase (ALP) (typical range: 200–750 U/L) and gamma-glutamyl transferase (GGT) (typical range: 100–400 U/L), and occasional elevation of alanine transaminase (ALT) (typical range: 0–200 U/L) and aspartate transaminase (AST) (typical range: 0–200 U/L) [2,18,23,32,44,45,49,50]. Higher levels of ALT and AST were observed in jaundiced patients [29]. Mild hyperbilirubinemia has been reported in both miliary and local hepatic TB cases [32]. Hepatic TB patients often had an inverted albumin to globulin ratio (A/G), in which the serum globulin was reported to be 1.25-1.86 times higher than serum albumin [14,18,20,45].

Laboratory abnormalities are generally similar between HIV-infected and -uninfected hepatic TB patients. In one small study, HIV-infected people had significantly higher levels of ALP (1,374.6 ± 714.4 U/L), as compared to HIV-uninfected people (472.2 ± 209.6 U/L) [45]. While abnormal liver function enzymes typically improve with treatment, in our review of 20 HIV-infected hepatic TB patients, we found normalization of liver enzymes lagged by months to years behind the clinical resolution [30].

### Diagnosis of hepatic tuberculosis

#### Clinical

A high index of clinical suspicion is required to make a diagnosis of hepatic TB. In TB-endemic regions, hepatic TB should be considered in patients who present with any combination of chronic right upper quadrant pain, hepatomegaly, fever, and weight loss [51]. An infiltrative pattern on liver function test with elevated ALP and GGT, as well as an inverted albumin/globulin ratio, would further support a clinical diagnosis of hepatic TB [2,14,18,20,23,32,44,45,49,50]. Where available, radiography and liver biopsy should be considered in addition to a clinical examination and laboratory tests.

#### Radiography

Plain x-ray radiography and ultrasound are generally the most widely available and first imaging tests to be obtained, but they both lack diagnostic specificity [15,20,52]. Plain abdominal radiography may show a focal hepatic lesion and/or hepatomegaly, but features may be similar to viral hepatitis, vascular disorders, systemic infections, other granulomatous diseases, or malignancy [15,52]. In one study, plain abdominal radiography revealed abnormalities in 86% (102/119) of cases, with diffuse hepatic calcifications in 49% (58/119) of patients [29]. A positive chest radiograph may also prompt additional investigations for hepatic TB, but a negative chest radiograph should not be used to exclude hepatic TB [18,20,29,44]. The median proportion of patients with abnormal chest radiographs was 42.5% (range: 0-88%) among hepatic TB case series (Table 4). Ultrasound has the benefit of being easily operated in TB-endemic resource-limited settings [53]. The median proportion of patients with abnormal abdominal ultrasound was 76% (range: 6-100%) among hepatic TB case series. However, abdominal ultrasound generally showed a round, non-specific hypoechoic region in the liver, and is therefore not an optimal test for hepatic TB [54,55]. Echogenic lesions of hepatic TB have also been described [56,57]. The primary value of plain radiographs or ultrasound would be to prompt further investigation and/or identify correct location for percutaneous liver biopsy [15,18,58,59].

**Table 4:** **Diagnostic findings in hepatic TB case series**

**Lead author**	**N**	**Imaging**			**Liver biopsy**		
**(Year)**		**ABN CXR (%)**	**ABN ABD US (%)**	**ABN ABD CT (%)**	**AFB+ smear (%)**	**Caseating granulomas (%)**	**PCR+ (%)**
Hersch (1964) [32]	143	ND	ND	ND	31 (11/35)	62 (89/143)	ND
Alvarez (1983) [29]	130	65 (83/128)	100 (16/16)	ND	7 (2/30)	68 (48/71)	ND
Essop (1984) [18]	96	75 (72/96)	ND	ND	9 (9/96)	83 (80/96)	ND
Maharaj (1987) [20]	41	78 (32/41)	6 (1/18)	ND	59 (24/41)	51 (21/41)	ND
Chien (1994) [2]	22	ND	ND	ND	18 (4/22)	86 (19/22)	ND
Kok (1999) [44]	5	0 (0/5)	100 (5/5)	100 (4/4)	ND	ND	ND
Huang (2003) [49]	5	20 (1/5)	40 (2/5)	40 (2/5)	20 (1/5)	80 (4/5)	100 (5/5)
Vilaichone (2004) [45]	20	ND	61 (11/18)&	ND	30 (6/20)	45 (9/20)	86 (12/14)
Yu (2004) [60]	12	17 (2/12)	25 (2/8)	100 (12/12)	ND	ND	ND
Desai (2006) [14]	7	0 (0/7)	100 (7/7)	100 (2/2)	43 (3/7)	14 (1/7)	ND
Amarakpurkar (2008) [76]	38	ND	79 (30/38)	88 (15/17)&	54 (21/39)	78 (18/23)	97 (28/29)
Tai (2008) [19]	10	ND	89 (8/9)	86 (6/7)	20 (2/10)	100 (10/10)	30 (3/10)
Hwang (2009) [50]	12	ND	ND	56 (5/9)	33 (2/6)	ND	75 (3/4)
Gounder (2012) [30]	20	88 (15/17)	73 (11/15)	ND	0 (0/14)	45 (9/20)^#^	ND
Median (Range)		42.5 (0–88)	76 (6–100)	88 (40–100)	25 (0–59)	68 (14–100)	86 (30–100)

TB = Tuberculosis; ABN = Abnormal; CXR = Chest x-ray; ABD = Abdominal; US = Ultrasound; CT = Computed tomography; AFB = Acid-fast bacilli; PCR = Polymerase chain reaction; ND = Not documented or not done, &Minimum abnormal, given documentation; ^#^Granulomas present, caseation not specified.

The optimal radiographic test for diagnosing hepatic TB is a contrast-enhanced abdominal CT scan or a dedicated triple-phase liver CT scan. The median proportion of patients with abnormal abdominal CT was 88% (range: 40-100%) among hepatic TB case series (Table 4). The CT findings are different for miliary and local hepatic TB. Miliary hepatic TB, which has smaller tubercles, is visualized on a CT scan as multiple, low-density micronodules dispersed throughout the liver [60]. CT imaging for miliary hepatic TB may also reveal hepatomegaly without nodular intrahepatic lesions, or it may reveal abdominal lymphadenopathy with peripheral lymph node enhancement and/or calcifications [59-61]. By contrast, local hepatic TB generally appears on CT as one large solitary nodule or 2–3 low-density nodules [54,60].

There were too few studies to recommend magnetic resonance imaging (MRI) as an appropriate imaging modality for hepatic TB, and the cost and accessibility of MRI scanning further limits its utility in TB-endemic resource-limited settings.

#### Liver biopsy

Liver biopsy with mycobacterial culture is considered the most specific diagnostic test for hepatic TB [18,51,32]. While liver biopsy may not always be necessary, microbiological and histological findings can allow for a more accurate diagnosis [11,24]. A liver biopsy is indicated in any person with a constellation of clinical, laboratory, and radiographic suspicion of hepatic TB [32,58]. These may include, but are not limited to, hepatomegaly of unknown origin, fever of unknown origin, and abnormal liver enzymes in any patient from a TB-endemic region [32,58,62]. A liver biopsy can be performed as a percutaneous transthoracic procedure along the mid-axillary line or as a subcostal abdominal procedure in patients with hepatomegaly [58]. Ultrasound guided liver biopsy is generally preferred to improve the sampling and increase the diagnostic accuracy [58,61]. The diagnostic yield of a liver biopsy may also be increased by using a large aspiration needle and performing more passes into the liver, but these should be weighed against the risk of complications [58].

Liver biopsies should be sent for both microbiological and histological evaluation. Microbiological methods include smear microscopy for acid-fast bacilli (AFB) and mycobacterial culture. AFB smear had a median sensitivity of 25% (range: 0-59%) among hepatic TB case series (Table 4). A positive AFB result may be from another *Mycobacterium* infection such as *Mycobacterium avium*, a common opportunistic infection of the liver in AIDS patients [63]. Mycobacterial culture provides the strongest evidence of hepatic TB, but the sensitivity has been reported to be <10% [64]. In our case series in South Africa, we noted positive culture in 36% (5/14) of patients [30]. Histological evidence of granulomas provides non-specific evidence for hepatic TB.^37^ Histological evidence of caseating granulomas had a median sensitivity of 68% (range: 14-100%) among hepatic TB case series. A diagnosis of hepatic TB based on hepatic granulomas may be supported by TB detected elsewhere in the patient [64]. In TB-endemic regions, the presence of a hepatic granuloma on biopsy should suggest a TB etiology, and may warrant a course of anti-TB therapy [11].

Since AFB smear and culture have low sensitivity and granulomas are not specific, nucleic acid amplification assays, such as polymerase chain reaction (PCR), have been recommended for diagnosing hepatic TB [64,65]. PCR had a median sensitivity of 86% (range: 30-100%) among hepatic TB case series (Table 4). In a series of 43 liver biopsies with granulomas, Diaz et al. used PCR to amplify the IS6110 insertion sequence to detect TB in 53% (9/17) of samples with confirmed or presumed TB and 4% (1/26) of samples without TB [64]. PCR testing had a sensitivity of 53%, specificity of 96%, and positive and negative predictive values of 90% and 76%, respectively. In another series of 35 patients, Alcantara-Payawal et al. used one-step and nested PCR to detect TB in liver biopsy samples with granulomas [65]. The sensitivity of one-step PCR was 100% (8/8) for the TB-confirmed group and 44% (4/9) for the presumed TB group, which then increased to 78% (7/9) using nested PCR. None of the 18 TB-negative controls tested positive for TB by PCR. These preliminary studies suggest that PCR may be more sensitive and specific for the diagnosis of TB in liver biopsy samples than AFB smear or culture [64,65]. In addition, PCR results can often be obtained earlier than mycobacterial culture results [64,65].

Another potential diagnostic tool is the Xpert MTB/RIF assay, a rapid nucleic acid amplification assay. The Xpert MTB/RIF assay has been approved for diagnosing pulmonary TB from sputum samples and is being evaluated for diagnostic accuracy in extrapulmonary tissues, including liver [66,67]. The Xpert assay can also provide a result in 2 hours and has the additional benefit of detecting rifampin (RIF) resistance mutations, a marker of multi-drug resistant TB [66]. As newer tests become more readily available, applying faster methods for diagnosing hepatic TB may promote early detection and prompt initiation of treatment.

A summary of the diagnostic criteria is provided in Table 5.

**Table 5:** **Traditional and updated diagnostic criteria for hepatic TB**

**Diagnostic criteria in 1984 [** 18 **]**	Updated diagnostic criteria in 2014*
• Acid fast bacilli on smear of liver tissue	• Culture of liver tissue demonstrating *M. tuberculosis*
• Culture of liver tissue demonstrating *M. tuberculosis*	• Acid fast bacilli on smear or nucleic acid (PCR) positive for TB (IS6110 insertion sequence) from liver tissue sample
• Caseating hepatic granulomata with a positive Mantoux reaction	• Abdominal CT demonstrating low-density hepatic nodule(s) (<2 mm: miliary; >2 mm: local) in patient with confirmed pulmonary TB or in a TB-endemic region
• Hepatic granulomata with demonstration of TB bacilli anywhere else in the patient	• Clinical presentation of right upper quadrant pain, hepatomegaly, fever, and weight loss in patient with confirmed pulmonary TB or in a TB-endemic region^**^
• Typical appearance at laparotomy	• Resolution of elevated liver enzymes following anti-TB therapy
• Autopsy confirmation of hepatic TB	
• Response to specific therapy	

TB = Tuberculosis; PCR = Polymerase chain reaction; CT = Computed tomography.

*Diagnostic criteria are presented from the strongest evidence to the weakest evidence.

**When available, other diagnostic tools such as radiography and liver biopsy should be employed to confirm clinical diagnosis.

### Treatment of hepatic tuberculosis

Anti-TB therapy should be initiated upon diagnosing hepatic TB, and considered in cases where clinical suspicion of hepatic TB is high [33]. Treatment of hepatic TB has been most successful with multi-drug regimens containing rifampin, isoniazid, and other anti-TB agents such as pyrazinamide, ethambutol, and/or streptomycin (Table 6). In one cohort of 96 hepatic TB patients, those receiving monotherapy had higher mortality (20% vs. 0%) compared to patients receiving a multi-drug regimen containing isoniazid and rifampin [18]. In another cohort, positive clinical responses, including improved appetite and reduced hepatomegaly, were seen in 67% (87/130) of patients receiving multi-drug therapy [29]. The WHO recommendation for the treatment of drug-susceptible pulmonary TB (rifampin, isoniazid, ethambutol, and pyrazinamide for two months, followed by 4 months of rifampin and isoniazid) [68,69] has been applied to hepatic TB with positive outcomes (Table 6). The optimal duration for treating hepatic TB is controversial, but 6–12 months appears to be effective for most patients (Table 6). The American Thoracic Society recommends 6–9 months for any extrapulmonary site, except the meninges [70]. Treatment has resulted in improved appetite and weight gain, resolution of fever, reduced jaundice, and decreased hepatosplenomegaly within the first two to three months [14,19,29,44]. Resolution of fever has been noted to occur within two weeks of initiating treatment, and appetite generally improved earlier [20].

**Table 6:** **Treatment regimens, outcomes, and mortality in hepatic TB case series**

**Lead Author (Year)**	**N**	**Treatment regimens (n)**	**Median duration (months)**	**Outcomes**	**Overall mortality (%)**	**Comments**
Alvarez (1983) [29]	130	H, S, E, PAS (130), biliary stenting (6)	ND	87 improved on H, S, E, PAS; 3 decompressed with stent	12	Author recommends treatment for 12 months
Essop (1984) [18]	96	<2 drugs (10); >2 drugs (no R, H) (29); R, H, +others (16); no TX (37); undocumented (4)	ND	52 improved; 40 died; 4 undocumented	42	No TX mortality: 95%; <2 drug mortality: 20%; R, H, +others mortality: 0%
Kok (1999) [44]	5	ATT (5)	ND	5 improved	0	All local hepatic TB
Huang (2003) [49]	5	ATT (3), lobectomy (1), hepatectomy (2); no TX (2)	ND	4 improved; 1 died	20	All local hepatic TB
Desai (2006) [14]	7	R, H, Z, E for 2 months, then R, H for 4 months (7)	6	6 improved; 1 died	14	All local hepatic TB
Amarapurkar (2008) [76]	38	R, H, Z, E for 2 months, then R, H for 10 months (38)	12	38 improved	0	None
Tai (2008) [19]	10	ATT (10), left hepatic resection (3)	≥6	9 improved; 1 LTFU	0	None
Hwang (2009) [50]	12	ATT (12)	12	10 improved; 2 had persistent disease	0	Disease persisted in patients w/ liver abscesses
Gounder (2012) [30]	20	R, H, Z, E (20)	9	12 improved; 8 died	40	2 patients died from MDR/XDR-TB

TB = Tuberculosis; H = Isoniazid; S = Streptomycin; E = Ethambutol; PAS = Paraaminosalicylic acid; ND = Not documented; R = Rifampin; TX = Treatment; ATT = Anti tuberculous therapy; Z = Pyrazinamide; LTFU = Lost to follow-up; MDR/XDR-TB = Multiple/extensively drug-resistant tuberculosis.

Monitoring hepatic TB patients is important to ensure response to treatment and detect complications. Hepatotoxicity, or drug-induced liver injury (DILI), is the most common adverse effect among patients receiving isoniazid, rifampin, and pyrazinamide [71,72]. However, the incidence and management of DILI among hepatic TB patients has been poorly studied. Managing hepatic TB in HIV co-infected patients involves additional challenges, and may require the assistance of an infectious diseases specialist. The WHO recommends initiating antiretroviral therapy (ART) 2–8 weeks after starting anti-TB therapy [69]. In addition to complex drug-drug interactions of concurrent ART and anti-TB therapy, clinicians should be aware of DILI and TB-related immune reconstitution inflammatory syndrome (TB-IRIS). Both entities can cause a worsening clinical picture and elevated liver enzymes after initiating ART, and can be difficult to distinguish [73]. TB-IRIS is an inflammatory reaction to TB antigens by the host immune system while receiving ART. Approximately one-sixth of co-infected patients starting ART will develop TB-IRIS [73]. Hepatic TB-IRIS presents with hepatomegaly (56% of patients), right upper quadrant pain, fever, nausea/vomiting, and elevated liver enzymes [73]. However, patients with hepatic TB-IRIS do not typically present with jaundice [73]. Differentiating DILI from hepatic TB-IRIS can be challenging in the absence of a liver biopsy [73]. Making this distinction is critical since DILI is managed with treatment cessation and drug rechallenge, while TB-IRIS requires persistence of treatment with the possible addition of corticosteroids [74,75].

## Conclusions

Hepatic TB is an extrapulmonary expression of active TB disease, and the incidence has likely increased during the era of HIV/AIDS, but definitive clinical studies are lacking. Clinicians in TB-endemic regions should have a high index of suspicion in patients presenting with hepatomegaly, fever, respiratory symptoms, and elevated liver enzymes. Abdominal radiography and ultrasound are non-specific imaging modalities, and a CT scan can be more accurate and should be preferred if available. Liver biopsy with mycobacterial culture and histology is the most specific test for diagnosing hepatic TB, but has poor sensitivity (or a high false negative rate). PCR of biopsy specimens has demonstrated high sensitivity and specificity for diagnosing hepatic TB. Patients with definitive or clinically suggestive hepatic TB should be promptly initiated on 4-drug anti-TB therapy, and clinicians should observe closely for drug toxicity and complications, such as DILI and TB-IRIS. Co-infection with HIV can complicate the management of hepatic TB, and clinicians must be knowledgeable of differences in pathophysiology, treatment, and disease management. A high index of suspicion for hepatic TB is important if clinicians are to make an early diagnosis and initiate prompt treatment to improve clinical outcomes.

## Abbreviations

TB: Tuberculosis
HIV: Human immunodeficiency virus
AIDS: Acquired immunodeficiency syndrome
CT: Computerized tomography
WHO: World Health Organization
ALP: Alkaline phosphatase
GGT: Gamma-glutamyl transferase
ALT: Alanine transaminase
AST: Aspartate transaminase
MRI: Magnetic resonance imaging
AFB: Acid-fast bacilli
PCR: Polymerase chain reaction
RIF: Rifampin
DILI: Drug-induced liver injury
ART: Anti-retroviral therapy
TB-IRIS: Tuberculosis-related immune reconstitution inflammatory syndrome

## References

[1] Bristowe JS (1858). On the connection between abscess of the liver and gastrointestinal ulceration. Transac Pathol Soc London..

[2] Chien RN, Lin PY, Liaw YF (1995). Hepatic tuberculosis: comparison of miliary and local form. Infection..

[3] World Health Organization. Tuberculosis: key facts. Reviewed Feb 2013. http://www.who.int/mediacentre/factsheets/fs104/en/.

[4] Glynn JR (1998). Resurgence of tuberculosis and the impact of HIV infection. Br Med Bull..

[5] Lawn SD, Zumla AI (2011). Tuberculosis. Lancet..

[6] Peto HM, Pratt RH, Harrington TA, LoBue PA, Armstrong LR (2009). Epidemiology of extrapulmonary tuberculosis in the United States, 1993–2006. Clin Infect Dis..

[7] Rieder HL, Cauthen GM, Bloch AB, Cole CH, Holtzman D, Snider DE (1989). Tuberculosis and acquired immunodeficiency syndrome. Arch Intern Med..

[8] Kwan CK, Ernst JD (2011). HIV and tuberculosis: a deadly human syndemic. Clin Microbiol Rev..

[9] Yang Z, Kong Y, Wilson F, Foxman B, Fowler AH, Marrs CF (2004). Identification of risk factors for extrapulmonary tuberculosis. Clin Infect Dis..

[10] Jones BE, Young SMM, Antoniskis D, Davidson PT, Kramer F, Barnes PF (1993). Relationship of the manifestations of tuberculosis to CD4 counts in patients with human immunodeficiency virus infection. Am Rev Respir Dis..

[11] Purl AS, Nayyar AK, Vij JC (1994). Hepatic tuberculosis. Indian J Tuberc..

[12] World Health Organization. Global tuberculosis report 2012. Reviewed Jun 2013. http://apps.who.int/iris/bitstream/10665/75938/1/9789241564502_eng.pdf.

[13] Mutreja D, Nangia R, Mishra P (2010). Pseudotumoral hepatic tuberculosis with pericardial abscess. Indian J Pathol Microbiol..

[14] Desai CS, Joshi AG, Abraham P, Desai DC, Deshpande RB, Bhaduri A (2006). Hepatic tuberculosis in absence of disseminated abdominal tuberculosis. Ann Hepatol..

[15] Arora R, Sharma A, Bhowate P, Bansal VK, Guleria S, Dinda AK (2008). Hepatic tuberculosis mimicking a Klatskin tumor: a diagnostic dilemma. Indian J Pathol Microbiol..

[16] Sarkar S, Saha K, Das CS (2010). Isolated tuberculous liver abscess in a patient with asymptomatic stage I sarcoidosis. Respir Care..

[17] Morris E (1930). Tuberculosis of the liver. Am Rev Tuberc..

[18] Essop AR, Posen JA, Hodkinson JH, Segal I (1984). Tuberculosis hepatitis: a clinical review of 96 cases. QJM..

[19] Tai WC, Kuo CM, Lee CH, Chuah SK, Huang CC, Hu TH (2008). Liver tuberculosis in Southern Taiwan: 15-years clinical experience. J Intern Med Taiwan..

[20] Maharaj B, Leary WP, Pudifin DJ (1986). A prospective study of hepatic tuberculosis in 41 black patients. QJM..

[21] Oliva A, Duarte B, Jonasson O, Nadimpalli V (1990). The nodular form of local hepatic tuberculosis: a review. J Clin Gastroenterol..

[22] Leeds IL, Magee MJ, Kurbatova EV, del Rio C, Blumberg HM, Leonard MK (2012). Site of extrapulmonary tuberculosis is associated with HIV infection. Clin Infect Dis..

[23] Wang JY, Hsueh PR, Wang SK, Jan IS, Lee LN, Liaw YS (2007). Disseminated tuberculosis: a 10-year experience in a medical center. Medicine..

[24] Monill-Serra JM, Martinez-Noguera A, Montserrat E, Maideu J, Sabate JM (1997). Abdominal ultrasound findings of disseminated tuberculosis in AIDS. J Clin Ultrasound..

[25] Orenstein MS, Tavitian A, Yonk B, Dincsoy HP, Zerega J, Iyer SK (1985). Granulomatous involvement of the liver in patients with AIDS. Gut..

[26] Sharma SK, Mohan A, Sharma A, Mitra DK (2005). Miliary tuberculosis: new insights into an old disease. Lancet Infect Dis..

[27] Wong EB, Omar T, Setlhako GJ, Osih R, Feldman C, Murdoch DM (2012). Causes of death on antiretroviral therapy: a post-mortem study from South Africa. PLoS One.

[28] Echejoh GO, Tanko MN, Manasseh AN, Ogala-Echejoh SE, Mandong BM, Okeke EN (2011). HIV/TB co-infection: liver biopsy findings. J Med Med Sci..

[29] Alvarez SZ, Carpio R (1983). Hepatobiliary tuberculosis. Digest Dis Sci..

[30] Gounder L, Drain PK, Hickey AJ, Moosa MYS (2012). Hepatic tuberculosis: A retrospective case–control study in Durban, South Africa.

[31] Culafic D, Boricic I, Vojinovic-Culafic V, Zdrnja M (2005). Hepatic tuberculomas: a case report. J Gastrointest Liver Dis..

[32] Hersch C (1964). Tuberculosis of the liver: a study of 200 cases. S Afr Med J..

[33] Chong VH, Lim KS (2010). Hepatobiliary tuberculosis. Singapore Med J..

[34] Turhan N, Kurt M, Ozderin YO, Kurt OK (2011). Hepatic granulomas: a clinicopathologic analysis of 86 cases. Pathol Res Pract..

[35] Dourakis SP, Saramadou R, Alexopoulou A, Kafiri G, Deutsch M, Koskinas J (2007). Hepatic granulomas: a 6-year experience in a single center in Greece. Eur J Gastroenterol Hepatol..

[36] Geramizadeh B, Jahangiri R, Moradi E (2011). Causes of hepatic granuloma: a 12-year single center experience from Southern Iran. Arch Iran Med..

[37] Drebber U, Kasper HU, Ratering J, Wedemeyer I, Schirmacher P, Dienes HP (2008). Hepatic granulomas: histological and molecular pathological approach to differential diagnosis – a study of 442 cases. Liver Int..

[38] Sabharwal BD, Malhotra N, Garg R, Malhotra V (1995). Granulomatous hepatitis: a retrospective study. Indian J Pathol Microbiol..

[39] Sanai F, Ashraf S, Ayman A, Sato MB, Batwa F, Al-Husseini H (2008). Hepatic granuloma: decreasing trend in a high incidence area. Liver Int..

[40] Onal IK, Ersoy O, Aydinli M, Yonem O, Harmanci O, Sokmenseur S (2008). Hepatic granuloma in Turkish adults: a report of 13 cases. Eur J Intern Med..

[41] Taori KB, Mahajan SM, Rewatkar A, Ghonge NP, Deshmukh AK, Mundhada RG (2003). Hepatic tuberculomas associated with fatty metamorphosis of liver: a case report. Indian J Tuberc..

[42] Brookes MJ, Field M, Dawkins DM, Gearty J, Wilson P (2006). Massive primary hepatic tuberculoma mimicking hepatocellular carcinoma in an immunocompetent host. MedGenMed..

[43] Coash M, Forouhar F, Wu CH, Wu GY (2012). Granulomatous liver diseases: a review. J Formos Med Assoc..

[44] Kok KYY, Yapp SKS (1999). Isolated hepatic tuberculosis: report of 5 cases and review of the literature. J Hepatobiliary Pancreat Surg..

[45] Vilaichone R, Mahachai V (2004). Hepatic tuberculosis: a clinico-pathological study. Thai J Gastroenterol..

[46] Lodenyo H, Segal I (2004). Hepatic disease in patients with acquired immunodeficiency syndrome (AIDS). Afr J Health Sci..

[47] Bendayan D, Litman K, Hendler A, Polansky V (2010). Liver tuberculosis in an HIV patient: diagnosis and management. Indian J Tuberc..

[48] Kielhofner MA, Hamill RJ (1991). Focal hepatic tuberculosis in a patient with acquired immunodeficiency syndrome. South Med J..

[49] Huang WT, Wang CC, Chen W, Cheng YF, Eng HL (2003). The nodular form of hepatic tuberculosis: a review with five additional new cases. J Clin Pathol..

[50] Hwang SW, Kim YJ, Cho EJ, Choi JK, Kim SH, Yoon JH (2009). Clinical features of hepatic tuberculosis in biopsy-proven cases. Korean J Hepatol..

[51] Alvarez SZ (1998). Hepatobiliary tuberculosis. J Gastroenterol Hepatol..

[52] Vimalraj V, Jyotibasu D, Rajendran, Ravichandran P, Jeswanth S, Balachandar TG (2007). Macronodular hepatic tuberculosis necessitating a hepatic resection: a diagnostic conundrum. Can J Surg.

[53] Heller T, Goblirsch S, Wallrauch C, Lessells R, Brunetti E (2010). Abdominal tuberculosis: sonographic diagnosis and treatment response in HIV-positive adults in rural South Africa. Int J Infect Dis..

[54] Levine C (1990). Primary macronodular hepatic tuberculosis: US and CT appearances. Gastrointest Radiol..

[55] Cao BS, Li XL, Li N, Wang ZY (2010). The nodular form of hepatic tuberculosis: contrast-enhanced ultrasonographic findings with pathologic correlation. J Ultrasound Med..

[56] Wetton CWN, McCarty M, Tomlinson D, Rosbotham J, Crofton ME (1993). Ultrasound findings in hepatic mycobacterial infections in patients with acquired immune deficiency syndrome (AIDS). Clin Radiol..

[57] Tan TC, Cheung AY, Wan WY, Chen TC (1997). Tuberculoma of the liver presenting as a hyperechoic mass on ultrasound. Br J Radiol..

[58] Grant A, Neuberger J (1999). Guidelines on the use of liver biopsy in clinical practice. Gut..

[59] Mortele KJ, Segatto E, Ros PR (2004). The infected liver: radiologic-pathologic correlation. Radio Graphics..

[60] Yu RS, Zhang SZ, Wu JJ, Li RF (2004). Imaging diagnosis of 12 patients with hepatic tuberculosis. World J Gastroenterol..

[61] Mert A, Ozaras R, Tabak F, Ozturk BM (2003). Localized hepatic tuberculosis. Eur J Intern Med..

[62] Ferrari TCA, Couto CM, Vilaca TS, Xavier MAP (2006). Localized hepatic tuberculosis presenting as fever of unknown origin. Braz J Infect Dis..

[63] Price JC, Thio CL (2010). Liver disease in the HIV-infected individual. Clin Gastroenterol Hepatol..

[64] Diaz ML, Herrera T, Lopez-Vidal Y, Calva JJ, Hernandez R, Palacios GR (1996). Polymerase chain reaction for the detection of Mycobacterium tuberculosis DNA in tissue and assessment of its utility in the diagnosis of hepatic granulomas. J Lab Clin Med..

[65] Alcantara-Payawal DE, Matsumura M, Shiratori Y, Okudaira T, Gonzalez R, Lopez RA (1997). Direct detection of Mycobacterium tuberculosis using polymerase chain reaction assay among patients with hepatic granuloma. J Hepatol..

[66] Vadwai V, Boehme C, Nabeta P, Shetty A, Alland D, Rodrigues C (2011). Xpert MTB/RIF: a new pillar in diagnosis of extrapulmonary tuberculosis?. J Clin Microbiol..

[67] Hillemann D, Rusch-Gerdes S, Boehme C, Richter E (2011). Rapid molecular detection of extrapulmonary tuberculosis by the automated GeneXpert MTB/RIF system. J Clin Microbiol..

[68] Centers for Disease Control. Diagnosis and management of mycobacterial infection and disease in persons with human immunodeficiency virus infection. Ann Intern Med. 1987;106:254–6. 10.7326/0003-4819-106-2-254 3800186

[69] World Health Organization (2010). Treatment of tuberculosis: guidelines - 4th edition.

[70] American Thoracic Society, CDC, Infectious Diseases Society of America. Treatment of tuberculosis. MMWR Recomm Rep. 2003;52(RR-11):1–77. 12836625

[71] Johnkennedy N, Abiodun AE, Ifeoma UH, Hope O, Ikechukwu NE, Nnedimma NC (2012). Alterations in some biochemical indices of hepatic function in tuberculosis patients on antituberclosis therapy. Indian J Med Healthc..

[72] Sonika U, Kar P (2012). Tuberculosis and liver disease: management issues. Trop Gastroenterol..

[73] Meintjes G, Sonderup MW (2011). A practical approach to the diagnosis and management of paradoxical tuberculosis immune reconstitution inflammatory syndrome. Contin Med Educ..

[74] Sharma SK, Singla R, Sarda P, Mohan A, Makharia G, Jayaswal A (2010). Saftey of 3 different reintroduction regimens of antituberculosis drugs after development of antituberculosis treatment-induced hepatotoxicity. Clin Infect Dis..

[75] Meintjes G, Scriven J, Marais S (2012). Management of immune reconstitution inflammatory syndrome. Curr HIV/AIDS Rep..

[76] Amarapurkar DN, Patel ND, Amarapurkar AD (2008). Hepatobiliary tuberculosis in western India. Indian J Pathol Microbiol..

